# Non-Sentinel Lymph Node Detection during Sentinel Lymph Node Biopsy in Not-Complete-Lymph-Node-Dissection Era: A New Technique for Better Staging and Treating Melanoma Patients

**DOI:** 10.3390/jcm10194319

**Published:** 2021-09-23

**Authors:** Franco Picciotto, Gianluca Avallone, Federico Castellengo, Martina Merli, Virginia Caliendo, Rebecca Senetta, Adriana Lesca, Désirée Deandreis, Maria Teresa Fierro, Pietro Quaglino, Simone Ribero

**Affiliations:** 1 Section of Surgical Dermatology, AOU Città Della Salute e Della Scienza, 10126 Turin, Italy; franco.picciotto@gmail.com (F.P.); virginia.caliendo@gmail.com (V.C.); 2 Department of Medical Sciences, Section of Dermatology, University of Turin, 10126 Turin, Italy; gianluca.avallone2@gmail.com (G.A.); fcastellengo@gmail.com (F.C.); merlimartina93@gmail.com (M.M.); mariateresa.fierro@unito.it (M.T.F.); pietro.quaglino@unito.it (P.Q.); 3 Pathology Unit, AOU Città Della Salute e Della Scienza di Torino, 10126 Turin, Italy; rebecca.senetta@unito.it; 4 Division of Nuclear Medicine, AOU Città Della Salute e Della Scienza, 10126 Turin, Italy; alesca@cittadellasalute.to.it (A.L.); desiree.deandreis@unito.it (D.D.)

**Keywords:** sentinel lymph node, non-sentinel lymph node, complete lymph node dissection, SPECT-CT, planar lymphoscintigraphy, melanoma, AJCC 8th classification

## Abstract

Sentinel lymph node biopsy has been demonstrated to be an effective staging procedure since its introduction in 1992. The new American Joint Committee on Cancer (AJCC) classification did not consider the lack of information that would result from the less usage of the complete lymph node dissection as for a diagnostic purpose. Thus, this makes it difficult the correct staging and would leave about 20% of the further positive non-sentinel lymph nodes in the lymph node basin. In this paper, we aim to describe a new surgical technique that, combined with single-photon emission computed tomography-computed tomography (SPECT-CT), allows for better staging of melanoma patients. This is a prospective study that includes 104 patients with cutaneous melanoma. Sentinel lymph node biopsy was offered according to the AJCC guideline. Planar lymphoscintigraphy was performed in association with SPECT-CT, identifying and removing all non-biologically “excluded” lymph nodes, guiding the surgeon’s hand in detection and removal of lymph nodes. Even if identification and removal of non-sentinel lymph nodes is unable to increase overall survival, it definitely gives better disease control in the basin. With a “classic” setting, the risk of leaving further lymph nodes out of the sentinel lymph node procedure is around 20%, thus, basically, the surgical sentinel lymph node of first and second lymph nodes would have therapeutic value and complete lymph node dissection classically performed.

## 1. Introduction

Sentinel node(s) is defined as any node on a direct lymphatic drainage pathway from the primary tumor. For the Sentinel lymph node biopsy (SLNB) procedure to be accurate, it is of critical importance that all true sentinel nodes are identified and harvested for examination. Therefore, seeing the lymphatic collectors enter the nodes by mean of dynamic phase of lymphoscintigraphy is vital to identify true sentinel node(s); single-photon emission computed tomography – computed tomography (SPECT-CT) enhanced lymphoscintigraphy can identify other unseen locations at planar lymphoscintigraphy (head/neck and trunk) and allow for the precise anatomical location of each SN to be determined, speeding the surgical retrieval. 

Sentinel lymph node biopsy (SLNB) has been demonstrated to be an effective staging procedure since its introduction in 1992 [1]. SLNB technique has been validated as a prognostic factor and, since the publication of the two papers (DECOG and MSLT-II) [2,3], it has become the “last” prognostic factor before making the decision on starting adjuvant treatment or to proceed with observation in Stage II–III melanoma. In fact, according to guidelines, it is not mandatory to perform complete lymph node dissection (CLND) after the results of these two studies, which have shown no advantages in melanoma disease specific survival whether SLNB positive patients receive immediate CLND or delayed after the evidence of nodal recurrence [4]. Moreover, the CLND carries a considerable burden of complications that is not a negligible aspect [5]. This has made SLNB the last surgical diagnostic procedure before the adjuvant therapy [4].

The new American Joint Committee on Cancer (AJCC) classification did not consider the lack of information that will result from less use of CLND for diagnostic purpose [6]. Thus, this makes correct staging difficult and would leave about 20% of the further positive non-sentinel lymph nodes (NSLNs) in the lymph node basin [7].

Lymph node drainage, since Halsted’s description, is a tree-shaped model. The first lymph node drains the specific melanoma site of the skin; a second level would derive from the first, and a third from the seconds. Moreover, in 10% of cases, the technique was unable to discriminate the first from the second lymph node in the basin, explaining why the majority of the case series reported an identification of an average of two SLNs with this technique [8].

In the SNLB standard technique, planar lymphoscintigraphy (PL) is used for sentinel lymph nodes (SLN) identification. Additionally, lymphoscintigraphy with single-photon emission tomography/computed tomography (SPECT/CT) could be usefully carried out to achieve better lymph node detection and anatomic localization [9].

When PL is performed in association with SPECT-CT, it provides a more precise localization of the sentinel lymph node compared to planar image only, defining anatomical and topographical details, even in the complex cervico-facial area. In fact, it is known that SNLB in head and neck melanoma is burdened by a higher false-negative rate than in other anatomical areas due to the unpredictability of lymphatic drainage, complex anatomy, and proximity of the sentinel lymph node to the tumor. In the head and neck, PL accurately documents the migration of the radioactive tracer but provides little information on the number and location of sentinel lymph nodes [10].

In this paper, we aim to describe a new surgical technique that, combined with SPECT-CT, allows for better staging of melanoma patients and, in the era of no-CLND, increases our capability of detecting even the second lymph node level.

## 2. Material and Methods

This is a prospective study that includes 104 patients with cutaneous melanoma. Sentinel lymph node biopsy was offered according to the AJCC guideline for all patients with a melanoma thicker than 0.8 mm (or less if ulcerated) [6], who accepted the diagnostic procedure and signed a proper consent form. In addition, T4 melanoma patients were submitted to the procedure according to the evidence [11]. All patients were submitted to preoperative ultrasound of the superficial basin and CT scan if Breslow thickness was above 2 mm, regardless of ulceration. In case of evidence of LN or visceral metastases, the patients were not included in the study since no indication to SLNB was given.

On the day before the surgery, 28.6 MBq of technetium 99 m labeled human serum albumin was injected into subdermal tissue in four sites on the edge of the primitive tumor. The radiopharmaceutical permit was obtained to perform a pre-operative planar lymphoscintigraphy (PL) and subsequently a SPECT-CT.

### SPECT-CT Technique

Preoperative and operative Technique SPECT-CT, performed in addition to PL, has the advantage of identifying and removing all lymph nodes that directly and/or indirectly drain the lymph from the primary tumor, to guide the surgeon’s hand in detection and removal of lymph nodes, resulting in a significant reduction in surgical time. The main limitation of this technique, in addition to the longer duration of the investigation and a limited increase in patient dosimetry for the use of low-dose CT, lies in the frequent impossibility of correctly defining the lymph nodes highlighted with SPECT-CT as “Sentinel Lymph Nodes” or “Non-Sentinel Lymph Nodes”; the involvement of these latter lymph nodes have a worse prognostic value [12] in the case of positivity to the histopathological examination—performed equally as sentinel lymph nodes (SNL) or indeterminable lymph nodes—by expert hands and in compliance with the Eight Edition of the American Joint Committee on Cancer (AJCC) guidelines [6]. In these situations, the surgeon must report to the pathologist all the lymph nodes for which it was not possible to recognize as belonging to either the sentinel or non-Sentinel group, labeling them as “indeterminable” [13].

Before the surgical intervention, a drawing was performed on the skin over the corresponding area of the first and eventually second SLNs detected with imaging techniques. In the operation theatre, the blue patent was injected in the site of the tumor and the surgical procedure was performed. 

A handheld gamma probe was used to identify the sentinel and non-sentinel lymph nodes. Furthermore, according to anatomical features of the patient, all the lymph nodes directly draining lymph from the tumor site—known as sentinel nodes—were excised regardless of their number. The first level II nodes directly draining from sentinel lymph nodes were then removed and classified as non-sentinel nodes. 

Time for the integrated SLNB procedure, including the careful dissection of the first NSLN identified by the combination of SPECT-CT and dynamic lymphoscintigraphy, is reported from the skin incision to the end of the stitching procedure.

## 3. Results

### 3.1. Patients

A total of 104 patients with melanoma were candidate to SLNB procedure: 50% were men (Table 1). On average, Breslow thickness was 2.58 mm and ranged from 0.6 to 10 mm. The 36% of primaries were ulcerated. The median number of mitosis was 2 (interquartile range IQR 1–5).

As to the primary site, 45 melanomas were located in the trunk (43%), 9 on the upper limbs (9%), 33 in the lower limbs (32%), and 17 on head and neck (16%). 

The association study showed that ulcerated melanomas are associated with greater thickness than non-ulcerated: (3.21 ± 2.45 vs. 1.48 ± 0.81, *p* < 0.01). An increased number of mitosis was associated with thicker melanomas and higher mitotic rate. 

### 3.2. SPECT-CT

**Figure 1:**
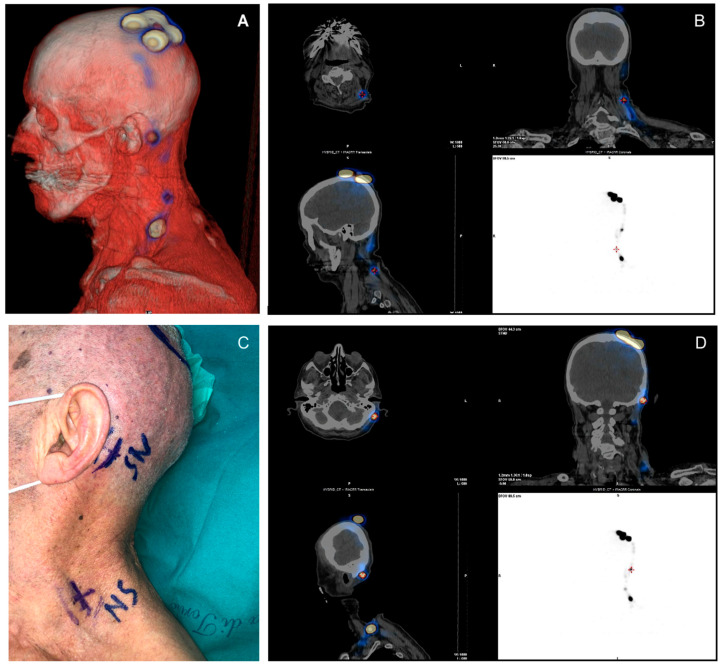
(Case 1): (**A**) A 3D image of the left retroauricular sentinel lymph node. (**B**–**D**) SPECT/CT rendering with a view of lymph nodes in CT reconstruction. (**C**) Preoperative image with skin marking of lymph nodes. SPECT-CT: single-photon emission computed tomography—computed tomography

**Figure 2:**
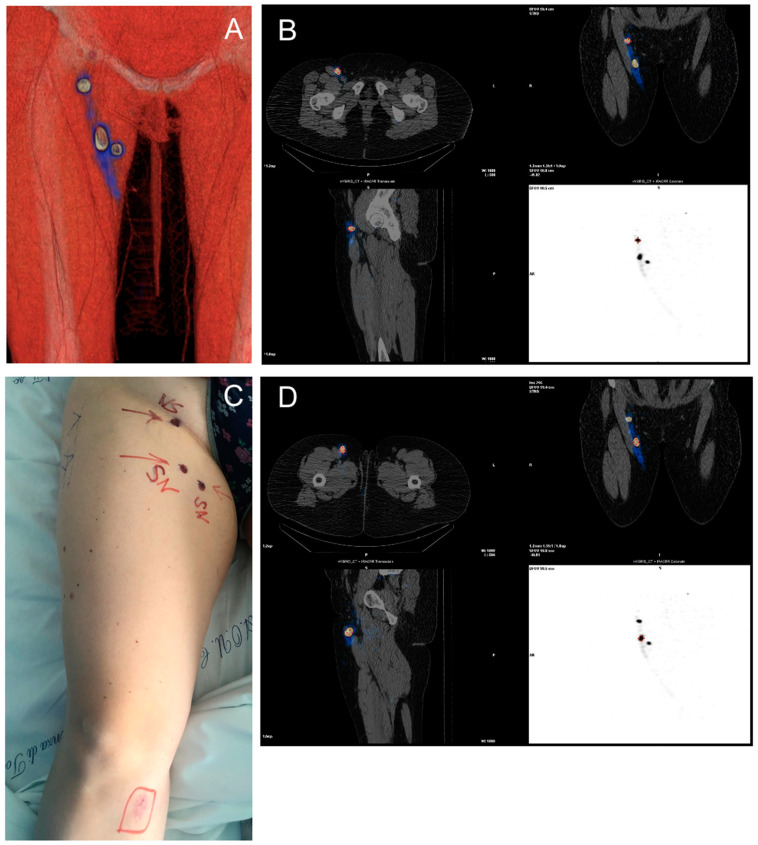
(Case 2) (**A**) A 3D image of the inguinal sentinel lymph node. (**B**–**D**) SPECT/CT rendering with a view of lymph nodes in CT reconstruction. (**C**) Preoperative image with skin marking of lymph nodes.

SPECT-CT was performed the day before surgery and allowed for the recognition of at least one sentinel lymph node in all patients (Figure 1A–D and Figure 2A–D). Fifteen cases had multiple basins involved (14.4%). In 49 cases the sentinel node was 1, in 39 cases there were 2, in 9 cases there were 3, in 5 cases there were 4, and in 2 cases there were 5.

### 3.3. SLNB Results

At least 1 positive SLN was found in 24 patients (23%), 2 positive SLN were found in 9, and 1 previous patient had 3 positive SLN out of 4 excised. 

The medium tumor burden, represented as the mean of the greatest dimension of the largest deposits, was equal to 2.6 mm. The median value was 2.25 mm (IQR: 1.5–4 mm).

### 3.4. NSLN Biopsy Results

**Figure 3:**
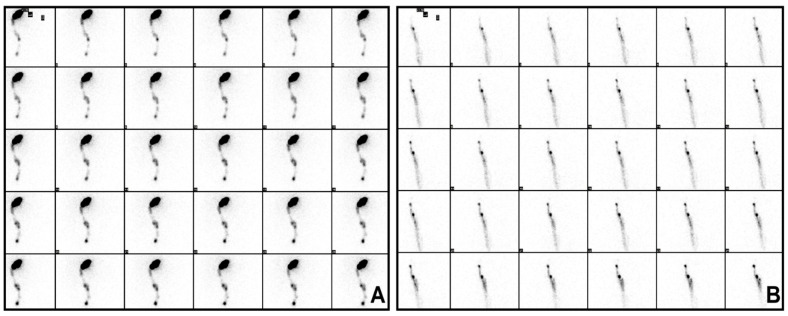
(**A**) Dynamic lymphoscintigraphy: single lymphatic pathway with one left retroauricular sentinel lymph node and two subsequent non-sentinel lymph nodes at the 5th left laterocervical level. (**B**) Dynamic lymphoscintigraphy: two lymphatic pathways with two adjacent sentinel lymph nodes (one medial and one lateral) right inguinal and one non-sentinel lymph node following the lateral pathway.

A careful dissection with the help of a lymphoscintigraphic dynamic phase (Figure 3A,B) plus the SPECT /CT permitted identification of 1 NSLN in 71 patients, 2 in 17, 3 in 10, and 4 in 2 patients. Only in 4 patients the technique did not allow detection of any NSLN. NSLN were in the same basin for all the patients; a part of the 5 cases were the only SLN in transit in the thoracic area and the NSLN in axilla, 1 case of a popliteal SLN and NSLN in inguinal basin, and in 1 case the SLN was in mastoid area and the NSLN in parotids. In 1 case, the SLN and the NSLN were both in transit in the thoracic wall. Among SLNB positive patients, histopathological analysis of NSLNs detected metastasis in 7 cases out of 24 positive SLN (29%). No patients presented negative SNL and positive NSLN status. 

The mean time for the procedure of SLN and NSLN detection was 83 ± 27 min; (79 min for axilla, 69 for groin, 116 cervical, 95 for bilateral, and 90 for unusual basins).

### 3.5. CLND

After multidisciplinary discussion, the complete lymph node dissection was performed depending on patients’ comorbidities, risk of disease progression needing further CLND and according to patients’ will not to undergo strict follow-up ultrasonographic examinations.

Thirteen SNL positive patients underwent the procedure, 4 of whom NSLN positive, showing metastatic involvement in 2 patients (1 metastatic lymph node out of 15 and 18 excised, respectively). In both cases there were positive NSLN, both in axilla of two different patients (10 mm and 12 mm, respectively, of Breslow thickness—one polypoid melanoma and one NM). Among NSLN positive patients, 3 did not undergo CNLD as requested by the patients. 

### 3.6. Medical Treatment and Follow-Up Data

Concerning adjuvant therapy, 5 out of 7 patients with NSLN involved are now under adjuvant therapy.

Both the patients with positive CLND are at the moment under adjuvant therapy, one presenting BRAF wild-type mutation with nivolumab and one BRAF V600E-mutated with dabrafenib plus trametinib.

After a median follow-up of 18 months (IQR 14–22), 3 patients relapsed in the same basin, two were previously submitted to CLND and 1 to adjuvant therapy; 2 patients (one receiving IFN and the other an instrumental follow up) of the same cohort have progressed in the visceral site (both at lung).

## 4. Discussion

In the “no CLND era”, for positive SLN patients a lower capability of staging and a risk of leaving metastatic nodes is higher than in the previous time. The latter, in particular, will make the potential therapeutical LN dissection more complicated and would increase the risk of surgical complications. The concept that NSLN are usually detected close to the SLN has been clearly reported and biologically fundamental [14].

PL is a very accurate technique in identifying the sentinel lymph node(s) in most patients with cutaneous melanoma, with a surgical detection rate of 95–98%. It is a consolidated technique, able to provide guidance for the surgeon by recognizing the lymphatic pathway that leads from the tumor site to the sentinel lymph node. The procedure requires a dynamic acquisition to track the transit of the radiotracer from the primary tumor to the SLN; the dynamic acquisition is followed by early static acquisitions and eventually delayed static acquisitions, including body regions of interest; the procedure can be completed by carrying out a whole-body scan (from the neck to the groin) [15,16]. However, the identification and surgical removal of the sentinel lymph node(s) may be conditioned by the absence of anatomical reference points and the limited spatial resolution of the planar scintigraphic examination. In fact, in some patients it is very difficult to determine the exact location of the sentinel lymph node, particularly if it is located deep or if it is located intrathoracically or intra-abdominally. 

The dynamic planar study, however, remains of fundamental importance in discriminating sentinel nodes from the non-sentinel nodes, owing to the visualization of the lymphatic pathways starting from the primary tumor site. This method, however, involves additional cost, more time for image acquisition, and exposure to a further dose of radiation (although CT is provided at low doses). Moreover, we developed a surgical technique that, with the help of SPECT-CT and a careful anatomic dissection of the lymph node basin, would be able to detect not only the first SLNs draining from the melanoma, but also the second, and distinguishing them, thus decreasing the possibility of leaving potential metastatic disease in the basin and providing further prognostic information. The technique does not increase significantly the surgical time setting.

A proper pre-operating nuclear medicine examination is fundamental in order to decrease the risk of false-negative SLN. The SLNB was considered false-negative if a primary recurrence developed in the regional lymph node basin from which a tumor-free SLN had been removed. Usually, the lymph node relapse appears after a median of 13 months from the SLN [17]. In this cohort, we assisted to 3 relapse out of 84 negative SLN (3%) with a false-negative rate of 13% (FN/FN + VP). These percentages are much less than those reported in literature [18,19].

In previous a case series from our clinic, we reported a NSLN positivity at the CLND of 31% [20]. Herein, applying the described technique with the use of SPEC/CT, 29% (7 out of 24 positive SLN patients) reported metastasis in the identified and excised NSLN. These percentages are almost superimposable and thus we can speculate that all the NSLN that would have been previously detected with the CLND are now able to be excised with the new technique, given an acceptable false-negative rate. We are conscious that the identification and removal of NSLN is unable to increase the overall survival (OS), but it definitely gives better disease control in the basin, especially in a historical moment when the CLND is no longer performed after a positive SLN. With a “classic” setting the risk of leaving further lymph nodes out of the SLN procedure is around 20%, so basically the surgical SLN of the first and second lymph nodes would have therapeutic value when the CLND is classically performed.

## 5. Conclusions

Even if identification and removal of non-sentinel lymph nodes is unable to increase overall survival, it definitely provides better control of disease in the basin. With a “classic” setting, the risk of leaving further lymph nodes out of the sentinel lymph node procedure is around 20%, so basically the surgical sentinel lymph node of first and second lymph nodes would have therapeutic value when the complete lymph node dissection is classically performed. 

## Figures and Tables

**Table 1:** Demographics and clinical characteristics of patients eligible for SNLB procedure.

		Patients (*n* = 104)
Sex (*n*)	F	52 (50%)
	M	52 (50%)
Age (years)	Mean	55.97
	Min–Max	22–77
Primary tumor site	Trunk	45 (43%)
	Head and neck	17 (16%)
	Upper limbs	9 (9%)
	Lower limbs	33 (32%)
Subtype	SSM	62 (59.62%)
	NM	24 (23.08%)
	LMM	1 (0.96%)
	ALM	4 (3.84%)
	Unclassified	13 (12.50%)
Tumor category	T1	28 (26.92%)
	T2	37 (35.58%)
	T3	20 (19.23%)
	T4	19 (18.27%)
Ulceration	Absent	67 (64.42%)
	Present	37 (35.58%)
Mitotic rate (mitoses per mm^2^)	<1	13 (12.50%)
	1	21 (20.19%)
	2–5	41 (39.42%)
	>5	29 (27.88%)
Breslow thickness (mm)		
	Mean	2.58
	Min–Max	0.6–10
Sentinel lymph nodes per patient (*n*)		
	1	49 (47.11%)
	2	39 (37.50%)
	3	9 (8.65%)
	4	5 (4.80%)
	5	2 (1.92%)
Positive sentinel lymph nodes		
	Yes	24 (23.07%)
	No	80 (76.93%)
Positive sentinel lymph nodes per patient (*n*)		
	1	14 (58.33%)
	2	9 (37.50%)
	3	1 (4.16%)
Diameter of sentinel lymph nodes metastasis (mm)		
	Mean	2.6
	Median	2.25
	Interquartile range	1.5–4
Non-sentinel lymph nodes per patient (*n*)		
	0	4 (3.84%)
	1	71 (68.26%)
	2	17 (16.34%)
	3	3 (2.88%)
	4	4 (3.84%)
Positive non-sentinel lymph nodes		
	Yes	7 (6.73%)
	No	97 (93.26%)
Complete lymph-nodes dissection		
	Yes	13 (12.50%)
	No	91 (87.50%)

SSM: superficial spreading melanoma; NM: nodular melanoma; LMM:lentigo maligna melanoma; ALM: acral lentiginous melanoma; SNLB: sentinel lymph nodes biopsy.

## Data Availability

All available information is contained within the manuscript.
